# New morphological and DNA evidence supports the existence of *Calligonum jeminaicum* Z. M. Mao (Calligoneae, Polygonaceae) in China

**DOI:** 10.3897/phytokeys.132.34981

**Published:** 2019-09-30

**Authors:** Wei Shi, Pei-Liang Liu, Jun Wen, Ying Feng, Borong Pan

**Affiliations:** 1 Key Laboratory of Biogeography and Bioresource in Arid Land, Xinjiang Institute of Ecology and Geography, Chinese Academy of Sciences, 830011, Urumqi, China Turpan Eremophytes Botanic Garden, Chinese Academy of Sciences Turpan China; 2 Turpan Eremophytes Botanic Garden, Chinese Academy of Sciences, 838008, Turpan, China Xinjiang Institute of Ecology and Geography Urumqi China; 3 College of Life Sciences, Northwest University, 710069, Xi’an, China Northwest University Xi'an China; 4 Department of Botany, National Museum of Natural History, Smithsonian Institution, 20013-7012, Washington DC, USA National Museum of Natural History, Smithsonian Institution Washington United States of America

**Keywords:** *Calligonum mongolicum* complex, Central Asia, desert plant, IUCN, molecular phylogenetics, morphological traits

## Abstract

*Calligonum jeminaicum* Z. M. Mao, a species regarded as endemic to China, was thought to be nonexistent owing to a lack of scientific records. The similarity of *C. jeminaicum* to *C. mongolicum* Turcz. warranted an investigation into the taxonomical relationship between these species. In this study, a naturally occurring population of *C. jeminaicum* was discovered and the taxonomical relationships of this species with *C. mongolicum* were resolved. Morphological traits, including fruit and flower characteristics, as well as nuclear (ETS, ITS) and chloroplast (*psbA-trnH*, *ycf6-psbM*, *rpl32-trnL*, *rbc*L, and *trnL-F*) DNA sequence data were studied to confirm the taxonomic status of *C. jeminaicum*. The nrDNA data (ITS1-2 and ETS) from *C. jeminaicum* reflected variability from the whole *C. mongolicum* complex, showing distinctive haplotypes in the Calligonum sect. Medusa Sosk. & Alexandr. The cpDNA data supplied similar evidence, showing unique branching in Bayesian and ML tree analyses. The specific status of *C. jeminaicum* is confirmed based on both morphological and molecular analyses. Here we present a revised description of *C. jeminaicum* along with its DNA barcode and discuss suggestions for the conservation of this species. Based on current evidence, this species was evaluated as Critically Endangered (CR) according to the IUCN criteria.

## Introduction

*Calligonum* L. species are as ecologically important as some of the dominant shrubs and semi-shrubs in both active and inactive sand dunes in the African Sahara (Dhief et al. 2011, 2012) and the deserts of Central Asia (Losinskaya 1927; Bao and Grabovskaya-Borodina 2003; Amirabadi-zadeh et al. 2012). They are natural resources of tannins, food, medications, nectar, and antidotes (Liu et al. 2001; Badria et al. 2007; Askariyahromi et al. 2013; Essam et al. 2014). *Calligonum* is considered to be the only genus within Polygonaceae that contains C_4_ species (Pyankov et al. 2000) and displays rapid rates of evolution and diversification (Mabberly 1990). This accelerated differentiation process causes physiological (Su et al. 2005, 2013) and morphological (Mao and Pan 1986; Taia and Moussa 2011; Tao and Ren 2004) changes within these species that facilitate their tolerance of various extreme xeric conditions (Pyankov et al. 2000; Su and Zhao 2002). Thus, *Calligonum* species have been used as the major sand conservation species in northwestern China (Wang et al. 2014; Xie et al. 2014).

*Calligonum jeminaicum* Z. M. Mao was first described by Mao (1984) to be a local endemic species which only proliferated in the countryside near Jeminay in the northwest of the Gurbantunggut Desert (Mao 1984, 1992). It has been difficult to differentiate *C. jeminaicum* from *C. mongolicum* Turcz. owing to their similar morphological characteristics (Mao 1992; Bao and Grabovskaya-Borodina 2003). In addition, there has been no further record of this species to demonstrate its existence, leading to the question: does this endemic species actually exist? This question was resolved by specific field work in 2013 when a naturally occurring population with eight individuals of *C. jeminaicum* was found.

**Figure 1.**
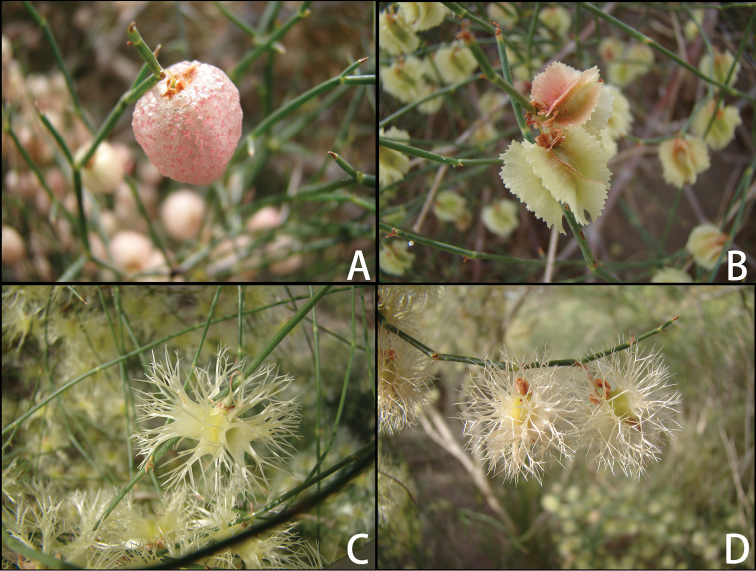
Fruit characters in the members of the four sections in *Calligonum* (A, sect. Calliphysa (Fisch. & C. A. Mey.) Borszcz.; B, sect. Pterococcus (Pall.) Borszcz.; C, sect. Calligonum ; D, sect. Medusa Sosk. & Alexandr.).

The rapid and complex evolutionary processes of *Calligonum* species have been reflected in their fruit morphology (Bao and Grabovskaya-Borodina 2003; Shi et al. 2009, 2016; Feng et al. 2010a; Soskov 2011). Fruit phenotype has been used as the key character to separate the whole Calligonum genus into four sections, namely sect. Calliphysa (Fish. & C. A. Mey.) Borszcz. (Fig. 1A), sect. Pterococcus (Pall.) Borszcz. (Fig. 1B), sect. Calligonum (Fig. 1C), and sect. Medusa Sosk. & Alexandr. (Fig. 1D). Members of sect. Calliphysa have membranous-saccate fruits, those of sect. Pterococcus have winged fruits, the fruits of sect. Medusa only show bristles without wings and membranes, and the fruits of sect. Calligonum show both wings and bristles but no membranes (Bao and Grabovskaya-Borodina 2003; Fig. 1). The most widely distributed species in Central Asia, *C. mongolicum* (sect. Medusa), has shown two karyotypes with different chromosome numbers (2*n* = 18 and 2*n* = 27) within the same population (Shi and Pan 2015); this species has heterogeneous phenotypes and forms a *C. mongolicum* complex with inter-crossed taxonomic relationships with other species in sect. Medusa (Soskov 2011; Shi et al. 2011, 2012; Shi and Pan 2015). *Calligonum mongolicum* has a large distribution area bordered by Xilinhot (Inner Mongolia) in the east, Kumul and Tutotu Basin (Xinjiang) in the west, Milan (Xinjiang) in the south, and Baitashan, Qitai, and Karamay (Xinjiang) in the north. The longitudinal range of *C. mongolicum* is about 30° (Pavlov 1936; Drobov 1953; Baitenov and Pavlov 1960; Sergievskaya 1961; Kovalevskaya 1971; Borodina 1989). The distribution range of *C. jeminaicum* lies within that of *C. mongolicum* (Mao 1992; Bao and Grabovskaya-Borodina 2003). The *C. mongolicum* complex has been the subject of several taxonomic studies, particularly those focused on species delimitation and identification (Feng et al. 2010b; Gulnur et al. 2010; Li et al. 2014; Shi et al. 2011, 2009, 2016). Both fruit and flower characteristics are used for distinguishing *C. jeminaicum* from *C. mongolicum* (Bao and Grabovskaya-Borodina 2003).

DNA analysis is regarded as one of the most important techniques to elucidate taxonomy (Kress et al. 2005; Hollingsworth et al. 2009). Previous studies have used *Calligonum* DNA data to resolve several conflicting taxonomic relationships, such as the use of RAPD markers to clarify the relationships of species in China (Ren et al. 2002), and the use of three chloroplast DNA markers (*rbcL*, *matK*, and *trnL-F*) to distinguish the Chinese species of *Calligonum*, although these conserved markers were not effective (Li et al. 2014). Additionally, cpDNA data have revealed the phylogeographic variation in different sections (Wen et al. 2015, 2016a, b), which was shown to be potentially valuable for DNA barcoding. ITS data have been used to effectively resolve taxonomical problems within the *C. mongolicum* complex (Shi et al. 2016, 2017). However, combined sequencing data from cpDNA and nrDNA have not been employed for clarifying the status of puzzling species in *Calligonum*. There is a need to further explore rapidly evolving DNA sequences that may be effective in resolving the taxonomic uncertainties in *Calligonum*.

In this study, nuclear ribosomal ITS and ETS sequences, together with five sets of cpDNA data (*psbA-trnH*, *ycf6-psbM*, *rpl32-trnL*, *rbcL*, and *trnL-F*) and the morphological characters, were used to confirm the existence of *C. jeminaicum* and clarify its relationship with *C. mongolicum*. We also suggest and discuss strategies for conserving *C. jeminaicum*.

## Methods

### Sample selection and species identification

All samples were collected from shoots of *Calligonum* individuals from Xinjiang, Qinghai, Inner Mongolia, Gansu, and Ningxia across the northwest of China during summer from 2006 to 2015 (Table 1).

**Table 1.** Population information for *C. mongolicum* Turcz., *C. jeminaicum* Z. M. Mao and related species, and GenBank accession numbers of DNA sequences used in this study.

Species	Pop. No. (#, &)^1^	Location	Latitude	Longitude	Elevation	Gen-Bank accession number							Voucher Number
						ITS	ETS	*psbA-trnH*	*trnL-trnF*	*ycf6-psbM*	*rpl32-trnL*	*rbcL*	
*C. mongolicum*	1(10, 4)	Erjinaqi, Inner Mongolia	41°27.2’N, 100°26.3’E		1002m	KU050846	KY316971	MN449309	MN449258	MN449070	MN449121	MN449172	C1101-C1110
						KU050848	KY316973	MN449310	MN449259	MN449071	MN449122	MN449173	
								MN449311	MN449260	MN449072	MN449123	MN449174	
								MN449312	MN449261	MN449073	MN449124	MN449175	
	2 (10, 2)	Hulishan, Inner Mongolia	41°58.3’N, 100°35.4’E		899m	MN449220	MN449032	MN449313	MN449262	MN449074	MN449125	MN449176	C1111-C1120
						MN449221	MN449033	MN449314	MN449263	MN449075	MN449126	MN449177	
	3 (10, 2)	Qingtongxia, Ningxia	38°01.0’N, 105°55.9’E		1134m	KU050847	KY316966	MN449315	MN449264	MN449076	MN449127	MN449178	C1121-C1130
						KU050853	KY316970	MN449316	MN449265	MN449077	MN449128	MN449179	
	4 (10, 3)	Mazongshan, Gansu	41°48.7’N, 098°42.4’E		12364m	MN449222	MN449034	MN449317	MN449266	MN449078	MN449129	MN449180	C1145-C1154
						MN449223	MN449035	MN449318	MN449267	MN449079	MN449130	MN449181	
						-	-	MN449319	MN449268	MN449080	MN449131	MN449182	
	5 (10, 2)	Liuyuan, Gansu	43°20.5’N, 091°23.6’E		1273m	KU050844	KY316963	MN449320	MN449269	-	MN449132	MN449183	C1166-C1175
						KU050845	KY316975	MN449321	MN449270	MN449081	MN449133	MN449184	
	6 (10, 3)	Kelamayi, Xinjiang	47°19.6’N, 086°46.4’E		574m	MN449224	MN449036	MN449322	MN449271	MN449082	MN449134	MN449185	C2101-C2110
						MN449225	MN449037	MN449323	MN449272	MN449083	MN449135	MN449186	
						-	MN449038	-	-	-	-	-	
	7 (10, 2)	Wuerhe, Xinjiang	46°08.2’N, 086°12.9’E		415m	KU050849	KY316969	MN449324	MN449273	MN449084	MN449136	MN449187	C2133-C2142
						KU050850	KY316972	MN449325	MN449274	MN449085	MN449137	MN449188	
	8 (10, 4)	Xinxinxia, Xinjiang	42°45.2’N, 095°28.7’E		1744m	MN449226	MN449039	MN449326	MN449275	MN449086	MN449138	MN449189	C2165-C2174
						MN449227	MN449040	MN449327	MN449276	MN449087	MN449139	MN449190	
						MN449228	MN449041	MN449328	MN449277	MN449088	MN449140	MN449191	
						MN449229	MN449042	MN449329	MN449278	MN449089	MN449141	MN449192	
	9 (10, 2)	Qijiaojing, Xinjiang	43°35.3’N, 091°25.4’E		1142m	KU050852	KY316960	MN449330	MN449279	MN449090	MN449142	MN449193	C2175-C2184
						KU050841		MN449331	MN449280	MN449091	MN449143	MN449194	
	10 (10, 3)	Hami1, Xinjiang	43°23.7’N, 091°32.5’E		1038m	-	-	MN449290	MN449239	MN449051	MN449102	MN449153	C2011-C2020
						KU050843	KY316962	MN449291	MN449240	MN449052	MN449103	MN449154	
						-		MN449292	MN449241	MN449053	MN449104	MN449155	
*C. mongolicum*	11 (10, 2)	Hami2, Xinjiang	42°44.5’N, 093°55.5’E		812m	MN449205	MN449019	MN449293	MN449242	MN449054	MN449105	MN449156	C2178-C2186
						MN449206	MN449020	MN449294	MN449243	MN449055	MN449106	MN449157	
	12 (10, 3)	Tashan, Xinjiang	45°01.7’N, 090°03.2’E		1018m	MN449207	MN449021	MN449295	MN449244	MN449056	MN449107	MN449158	C2274-C2283
						MN449208	-	MN449296	MN449245	MN449057	MN449108	MN449159	
						MN449209	-	MN449297	MN449246	MN449058	MN449109	MN449160	
	13 (10, 2)	Chaidamu, Qinghai	39°09.7’N, 089°47.4’E		1680m	MN449210	MN449022	MN449298	MN449247	MN449059	MN449110	MN449161	C0121-C0130
						-	-	MN449299	MN449248	MN449060	MN449111	MN449162	
	14 (10, 3)	Kumishi, Xinjiang	42°14.5’N, 088°13.4’E		919m	MN449211	MN449023	MN449300	MN449249	MN449061	MN449112	MN449163	C0152-C0161
						MN449212	MN449024	MN449301	MN449250	MN449062	MN449113	MN449164	
						MN449213	MN449025	MN449302	MN449251	MN449063	MN449114	MN449165	
	15 (10, 1)	Heshuo, Xinjiang	42°16.9’N, 082°59.2’E		1105m	MN449214	MN449026	MN449303	MN449252	MN449064	MN449115	MN449166	C0122-C0131
	16 (10, 3)	Mingfeng, Xinjiang	36°45.1’N, 082°59.3’E		1600m	MN449215	MN449027	MN449304	MN449253	MN449065	MN449116	MN449167	C0174-C0184
						MN449216	MN449028	MN449305	MN449254	MN449066	MN449117	MN449168	
						MN449217	MN449029	MN449306	MN449255	MN449067	MN449118	MN449169	
	17 (10, 2)	Yutian, Xinjiang	36°45.2’N, 082°02.1’E		1648m	MN449218	MN449030	MN449307	MN449256	MN449068	MN449119	MN449170	C0147-C0158
						MN449219	MN449031	MN449308	MN449257	MN449069	MN449120	MN449171	
*C. jeminaicum*	18 (8, 3)	Jeminay, Xinjiang	47°19.3’N, 086°45.9’E		780m	MN449232	MN449048	MN449334	MN449283	MN449094	MN449146	MN449197	C3225-C3233
						MN449233	MN449049	MN449335	MN449284	MN449095	MN449147	MN449198	
						MN449234	MN449050	-	-	-	-	-	
*C. calliphysa*	19 (0, 1)	Mulei, Xinjiang	44°35.8’N, 090°39.7’E		574m	KX186585	KY316976	MN449338	MN449287	MN449099	MN449150	MN449202	C0112-C0121
	20 (0, 1)	Qitai, Xinjiang	44°59.4’N, 089°57.5’E		540m	KX186585	KY316976	MN449339	MN449288	MN449100	MN449151	MN449203	C2301-C2310
*C. ebinuricum*	21 (0, 3)	Jinhe, Xinjiang	44°37.8’N, 083°11.1’E		370m	MN449236	MN449045	MN449336	-	MN449096	MN449148	MN449199	C1158-C1167
						MN449237	MN449046	MN449337	MN449285	MN449097	MN449149	MN449200	
						MN449238	MN449047	-	MN449286	MN449098	-	MN449201	
*C. arborescens*	22(0, 2)	Huocheng, Xinjiang	44°4.58’N, 080°29.2’E		639m	MN449230	MN449043	MN449332	MN449281	MN449092	MN449144	MN449195	C1168-C1177
						MN449231	MN449044	MN449333	MN449282	MN449093	MN449145	MN449196	
*Pteroxygonum giraldii*	23(0,1)	Ningshan, Shaanxi	33°48.5’N, 108°39.7’E		1501m	MN449235	-	MN449340	MN449289	MN449101	MN449152	MN449204	P. L. Liu 431

^1^ #, the number of samples used for morphological analysis; &, the number of samples used for DNA analysis

**Figure 2.**
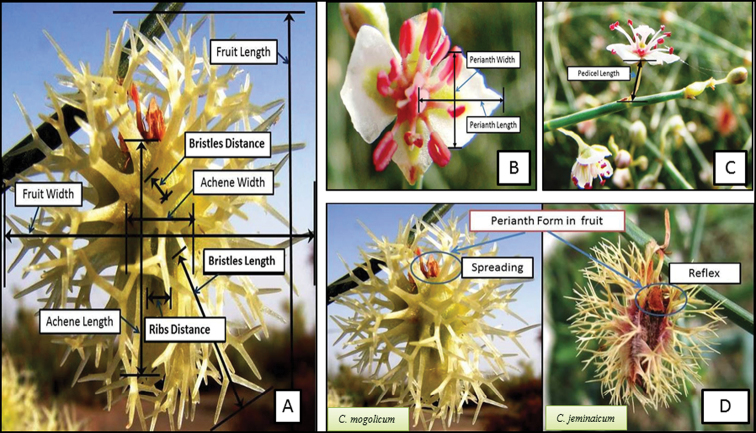
Measurements of fruit characters (**A**) and flower traits (**B** form of perianth segments **C** pedicel), and the distinction of the form of perianth segments in *Calligonum jeminaicum* and *C. mongolicum* fruits (**D**).

The classical identification key was used to differentiate these species mainly based on fruit characteristics and geographic locations, and the *C. mongolicum* complex has been identified by its fruit characteristics (Mao 1992; Bao and Grabovskaya-Borodina 2003), primarily based on quantifiable differences in fruit and bristle size, such as fruit length (LF), fruit width (WF), bristle length (BS), bristle distance (BD), rib distance (RD), achene length (AL), achene width (AW), and fruit shape (FF) (Shi et al. 2012, 2016; Fig. 2A). The same fruit indices have been used to compare *C. jeminaicum* with *C. mongolicum*. The flower traits for differentiating between the two species were selected based on the identification key in “Flora of China” (Bao and Grabovskaya-Borodina 2003), including the shape of perianth segments (PS, broadly elliptic or ovate Fig. 2B), pedicel length (1–2 cm in *C. jeminaicum* and 2–4 cm in *C. mongolicum*: Fig. 2C), spreading or reflexed in fruit (PSF, Fig. 2D), and pedicel joint position (below or middle). The shape of perianth segments (Fig. 2B) and pedicel length (Fig. 2C) were used to make quantitative distinctions between *C. jeminaicum* and *C. mongolicum*.

Some species with distinctive fruit characters were used as references in the DNA data analysis: *Calligonum calliphysa* Bunge, which was previously named *Calligonum junceum* (Fisch. & C. A. Mey.) Litv. (Bao and Grabovskaya-Borodina 2003), is the only species in sect. Calliphysa, was selected as a representative species; *Calligonum arborescens* Litv. and *Calligonum ebinuricum* Ivanova ex Y. D. Soskov (sect. Medusa) were used for comparison because they are regarded as distinct from the *C. mongolicum* complex. The number of individuals used for morphological analysis and DNA extraction in each population and the accession numbers of some ITS and plastid marker sequences obtained from GenBank are given in Table 1.

### Molecular protocols

For all the newly collected samples, total genomic DNA was extracted from fresh or silica gel dried leaves according to the protocol of Doyle and Doyle (1990) or the CTAB method of Doyle and Doyle (1990). The ribosomal DNA regions are known to be potentially problematic when inferring phylogeny (Alvarez and Wendel 2003). In this study, we followed the guidelines for obtaining reliable ITS sequences in plants proposed by Feliner and Rossello (2007). The ITS regions were amplified and sequenced using the previously described primers “ITS5a” and “ITS4” (Stanford et al. 2000). The ETS primers were designed by Shi et al. (2016): the forward primer ETScalli1: 5'-GTTACTTACACTCCCCACAACCCC-3' and the reverse primer 18SIGS: 5'-GAGACAAGCATATGACTACTGGCAGGATCAACCAG-3'. Primers and polymerase chain reaction (PCR) protocols used for the amplification of chloroplast *psbA-trnH*, *ycf6-psbM*, *rpl32-trnL*, *trn*L-F, and *rbc*L (the first part of the entire *rbc*L gene) were described in previous studies (Demesure et al. 1995; Small et al. 1998; Shaw et al. 2005, 2007; Falchi et al. 2009).

The specific Sanger sequencing studies of the *Calligonum mongolicum* complex and other species were divided into two parts, with most experiments completed at the Smithsonian Institution in 2014, and additional data, particularly those concerning *C. jeminaicum*, being supplied by the Key Laboratory of Biogeography and Bioresource in Arid Land (KLBB), Xinjiang Institute of Ecology and Geography, Chinese Academy of Sciences. At the Smithsonian Institution, PCR amplification of DNA was performed using 10 ng of genomic DNA, 4 pmol of each primer, 0.5 U Taq polymerase (Bioline, Randolph, MA, USA), and 2.5 mM MgCl_2_ in a volume of 25 µL using a PTC-225 Peltier thermal cycler. The PCR cycling parameters were as follows: a 95 °C initial hot start for 5 min, 32 cycles of 94 °C for 30 s, primer-specific annealing (ITS and ETS: 55 °C for 60 s; the five cpDNA primers: 53 °C for 40 s), and 72 °C for 60 s, and a final extension of 72 °C for 10 min. At the Smithsonian Institute, the PCR products were isolated and purified using ExoSAP-IT (US Biological, Swampscott, MA, USA) and sequenced in both directions using the PCR primers. Cycle sequencing was carried out using an ABI Prism Big Dye Terminator Cycle Sequencing Ready Reaction kit (Applied Biosystems, Foster City, CA, USA) with 5 ng of each primer, 1.5 µL of sequencing dilution buffer, and 1 µL of cycle sequencing mix in a 10 µL reaction volume. Cycle sequencing conditions comprised 30 cycles of 30 s denaturation (96 °C), 30 s annealing (50 °C), and 4 min elongation (60 °C). The sequencing products were separated on an ABI 3730xl DNA analyzer (Applied Biosystems, Foster City, CA, USA). At KLBB, the amplified products were purified using a PCR Product Purification Kit (Shanghai SBS, Biotech Ltd., China). Sequencing reactions were conducted with the forward and reverse PCR primers using the DYEnamic ET Terminator Cycle Sequencing Kit (Amersham Biosciences, Little Chalfont, Buckinghamshire, U.K.) with an ABI PRISM 3730 automatic DNA sequencer (Shanghai Sangon Biological Engineering Technology & Services Co., Ltd., Shanghai, China). Both strands of the DNA were sequenced with overlapping regions to ensure that each base was unambiguous. Electropherograms were assembled and consensus sequences were generated with Sequencher 4.5 (Gene Codes, Ann Arbor, MI, USA).

### Phylogenetic and network analyses

Multiple sequence alignments were performed using MUSCLE in the Geneious v.10.0.6 platform (Kearse et al. 2012) using the default settings and manual adjustments. The phylogenetic tree reconstruction of the nrITS and ETS sequence alignment included 44 accessions: 35 newly generated nrITS sequences, 24 new ETS sequences, and nine ITS and 20 ETS sequences from GenBank (Table 1).

Phylogenetic analyses were conducted on both the nuclear and combined plastid datasets. The best-fit nucleotide substitution models for the ITS1, 5.8S, ITS2, ETS, *psbA-trnH*, *ycf6-psbM*, *rpl32-trnL*, *trnL-F*, and *rbcL* regions were determined separately using jModelTest (Darriba et al. 2012) and the Akaike information criterion (AIC) were used to rank the best-fit model for the Bayesian analyses.

Phylogenetic relationships were inferred using Bayesian inference (BI) as implemented in MrBayes v.3.2.5 (Ronquist and Huelsenbeck 2003) and the maximum likelihood (ML) analyses were accomplished with RAxML v.8.2 (Stamatakis 2014). Partitioned analyses of both the nuclear and plastid datasets were implemented by applying the previously determined models to each data partition (Brown and Lemmon 2007). The nuclear ITS dataset was partitioned into ITS1, 5.8S, and ITS2 partitions. For the concatenated plastid dataset, separate partitions were used for the *psbA-trnH*, *ycf6-psbM*, *rpl32-trnL*, *trnL-F*, and *rbcL* regions. 51 samples in *Calligonum* were selected as the ingroup and *Pteroxygonum giraldii* Dammer & Diels was selected as the outgroup. Two independent BI analyses with one cold and three incrementally heated Markov chain Monte Carlo (MCMC) chains were run for 10,000,000 generations, with trees sampled every 1,000 generations. All Bayesian analyses produced split frequencies of less than 0.01, indicating convergence between the paired runs. The first 2,500 trees were discarded as burn-in, and the remaining trees were used to construct a 50% majority-rule consensus tree and posterior probabilities (PP). In the ML analyses, rapid bootstrap analysis was performed with a random seed, 1,000 alternative runs, and the same partition scheme as was used in the Bayesian analysis. The model parameters for each partition of the dataset were optimized by RAxML with the GTRCAT command. Trees were visualized in FigTree v1.4.3 (http://tree.bio.ed.ac.uk/software/figtree/). The ML bootstrap support values (BS) were labeled on the corresponding branches of the BI trees.

A network analysis was carried out with SplitsTree 4.13.1 (Huson and Bryant 2006) using the uncorrected *p*-distances between the *C. mongolicum* complex and *C. jeminaicum* species from the Bayesian analyses. Branch support was estimated using bootstrapping with 1,000 replicates (Felsenstein 1985).

## Results

### Phenotyping

The descriptions of the shape of perianth segments in fruit (PSF) and the pedicel joint position (below or middle) used to distinguish between the two species were qualitatively compared. The shape of perianth segments in fruit differs between the two species: spreading in the fruit of *C. mongolicum*, but reflexed in that of *C. jeminaicum* (Fig. 2D).

**Figure 3.**
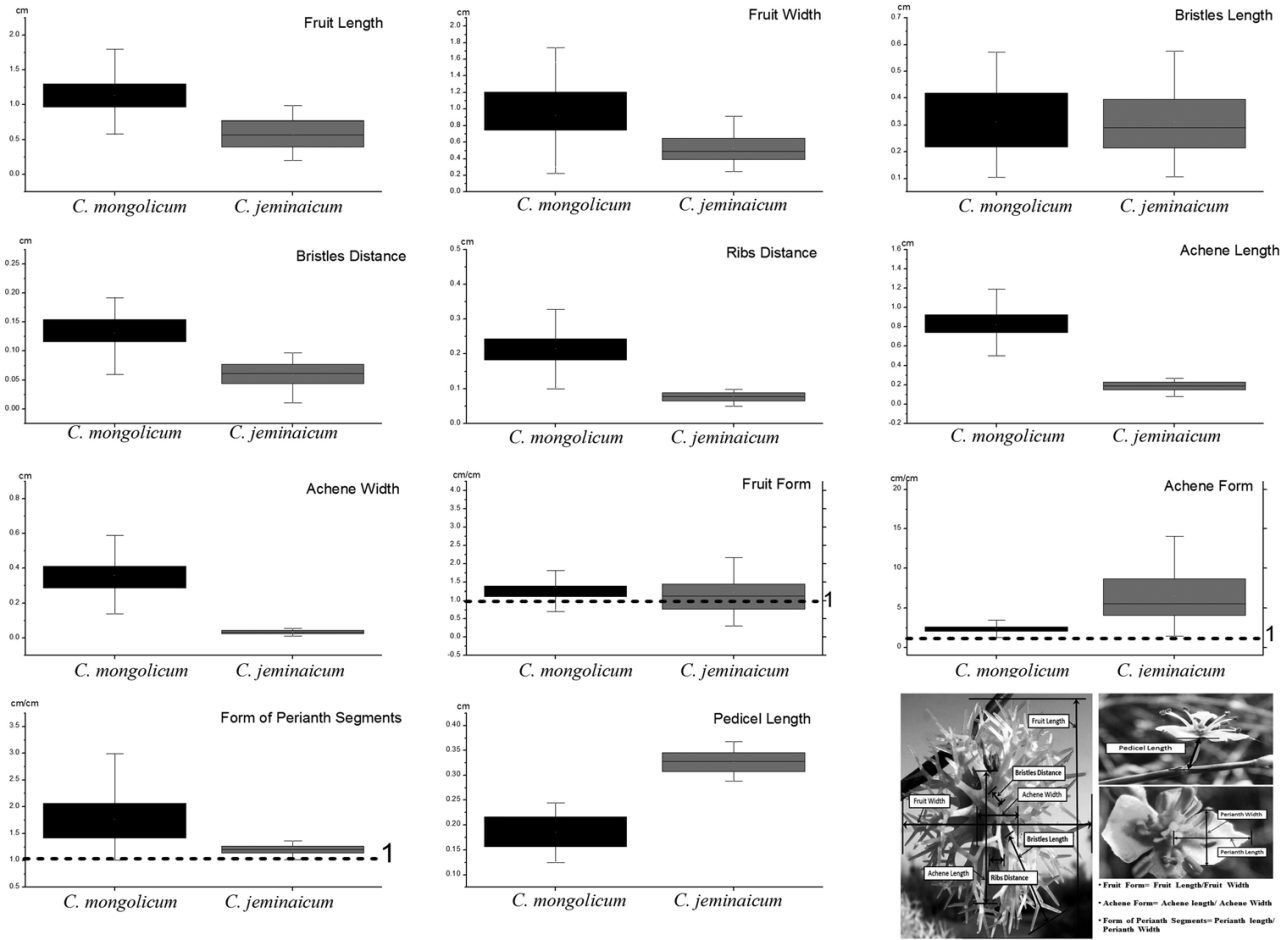
Quantitative comparisons of fruit and flower characters in *Calligonum mongolicum* and *C. jeminaicum*.

The morphological differences between *C. mongolicum* and *C. jeminaicum* focus primarily on their fruit and flower characteristics. Compared with the ambiguous characters in *C. mongolicum*, these taxonomical characters of *C. jeminaicum* were clearer and more stable. Quantitative comparisons of the fruit traits (Fig. 2A), the perianth segment shape (broadly elliptic or ovate, identified by the value of the length of the perianth segments/width of the perianth segments: Fig. 2B), and the pedicel length (Fig. 2C) were made between the two species (Fig. 3). Although some fruit characters appeared simultaneously in the two species and led to difficulty in distinguishing *C. jeminaicum* from *C. mongolicum*, the shape of perianth segments in fruit could be regarded as an effective character for their identification (Fig. 2D).

The quantifiable morphological characters in both fruits and flowers were compared between the two species. The fruit of *C. mongolicum* (0.106–1.880 cm; 1.134 ± 0.284 cm) was significantly (*P* = 0.026) longer than that of *C. jeminaicum* (0.415–0.649 cm; 0.432 ± 0.44 mm). Additionally, the fruit width (FW) for *C. mongolicum* (0.226–1.742 cm; 0.923 ± 0.347 cm) was larger than that of *C. jeminaicum* (0.348–0.508 cm; 0.428 ± 0.113 cm; *P* = 0.017). The bristle length of *C. jeminaicum* (0.372 ± 0.020 cm) was significantly shorter (*P* = 0.06) than that of *C. mongolicum* (0.312 ± 0.121 cm), and the bristle distance (0.077 ± 0.006 cm) and rib distance (0.087 ± 0.004 cm) of *C. jeminaicum* were significantly smaller than those of *C. mongolicum* (bristle distance 0.131 ± 0.032 cm, *P* = 0.01; rib distance 0.105 ± 0.032 cm, *P* = 0.02). Significant differences were also detected in achene length (0.823 ± 0.146 cm in *C. mongolicum* and 0.195 ± 0.105 cm in *C. jeminaicum*, *P* = 0.00) and achene width (0.359 ± 0.089 cm in *C. mongolicum* and 0.333 ± 0.004 cm in *C. jeminaicum*, *P* = 0.00) (Fig. 3), although the difference in achene width was small. The fruit shape, as the key character, was substantially different between the two species (*P* = 0.000), with the subglobose fruit of *C. jeminaicum* (1.048 ± 0.467 cm/cm) being much more rounded than the broadly ellipsoid fruit of *C. mongolicum* (1.357 ± 0.442 cm/cm). Thus, the fruit characteristics could be used to distinguish between the two species (Fig. 3). Both the pedicel length (*P* = 0.00) and the form of perianth segments (*P* = 0.01) of the two species showed significant differences. The pedicel length of *C. jeminaicum* (0.313 ± 0.004 cm) was much longer than that of *C. mongolicum* (0.219 ± 0.03 cm). The shape of perianth segments for *C. jeminaicum* (1.222 ± 0.167 cm/cm) was broader than that of *C. mongolicum* (2.544 ± 1.799 cm/cm) (Fig. 3).

### Molecular phylogeny

The aligned matrix of 44 accessions of the combined nrITS and ETS sequences comprised 807 bp that did not include any abnormal SNPs or unreasonable sequences according to the Phi test (*P* = 0.0321). The best-fit substitution models were GTR+G for ETS (nucleotide frequencies A: 0.200803 C: 0.329510 G: 0.295074 T: 0.174613) and GTR+I+G for nrITS (nucleotide frequencies A: 0.163227 C: 0.337699 G: 0.352720 T: 0.146353) based on the jModelTest (Darriba et al. 2012) results. The GTR+G model was selected for the ML analyses of the aligned matrix of nrDNA.

**Figure 4.**
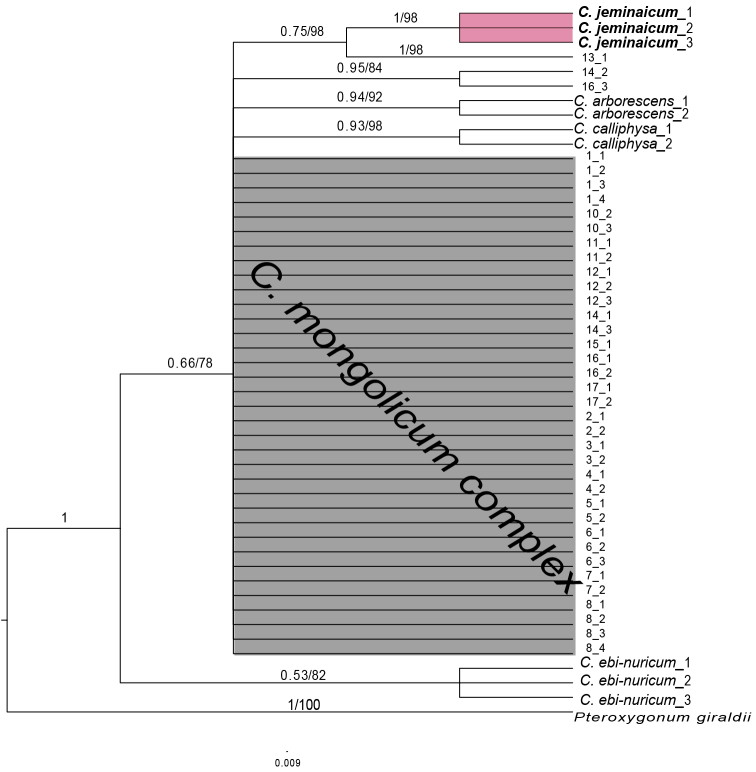
Bayesian inference tree of the concatenated nuclear ITS and ETS sequence data showing *Calligonum jeminaicum* and its congeners. Bayesian posterior probabilities and maximum likelihood bootstrap support values are given above the branches.

**Figure 5.**
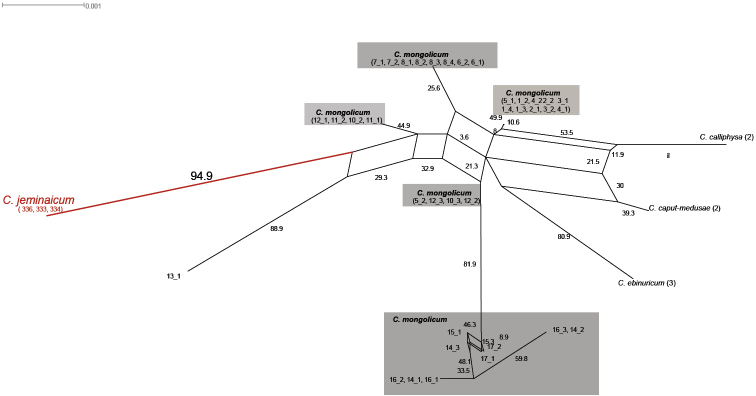
Neighbor-net analyses based on uncorrected p-distances of the nuclear ITS and ETS sequence data. Numbers indicate bootstrap values over 1,000 replicates.

**Figure 6.**
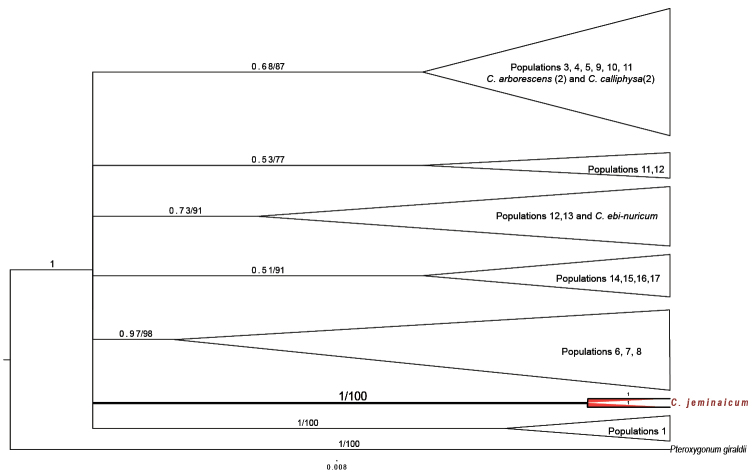
Bayesian inference tree of the concatenated plastid DNA sequence data (*psbA-trnH*, *ycf6-psbM*, *rpl32-trnL*, *rbcL*, and *trnL-F*) showing *Calligonum jeminaicum* and its congeners. Bayesian posterior probabilities and maximum likelihood bootstrap support values are given above the branches.

The two phylogenetic tree reconstruction methods, BI and ML, produced consistent topologies. However, the nuclear and the chloroplast data were analyzed separately to reconstruct the phylogenetic relationships among *C. jeminaicum*, the *C. mongolicum* complex, and other species in *Calligonum* because obviously different topologies based on the nuclear (Fig. 4, 5) and the chloroplast (Fig. 6) data were found. In the nrDNA data, no single nucleotide polymorphism (SNP) was identified among the *C. jeminaicum* samples, but the species from the *C. mongolicum* complex showed heterogeneity and did not form a single clade (Fig. 4). The populations of the *C. mongolicum* complex, *C. arborescens*, *C. calliphysa*, and *C. jeminaicum*, were distributed within the same broad geographic region. The three individuals of *C. ebinuricum*, which had specific fruit characteristics that were different from the *C. mongolicum* complex, formed an independent clade (Fig. 4). Interestingly, the *p*-distance among the *Calligonum* taxa for the ITS and ETS regions reached 11.364% between species *C. arborescens* and *C. calliphysa*. The *p*-distance was as high as 22.54% between *C. ebinuricum* and the *C. mongolicum* complex group, which reflects their interspecific differentiation. Consistent results were obtained in the ML analysis in the same phylogenetic tree for nrDNA, conforming the *C. mongolicum* complex and *C. jeminaicum* independently (Fig. 4, PP = 1, BS = 98%).

The neighbor-net constructed for the *C. mongolicum* complex and *C. jeminaicum* using the ITS and ETS sequences (Fig. 5) also did not support a single clade for the *C. mongolicum* complex. The three *C. jeminaicum* samples formed a separate branch from other groups, which is distant from the entire *C. mongolicum* complex, with a bootstrap support value of 94.9%.

Independent phylogenetic trees were reconstructed based on the concatenated plastid dataset, including the *psbA-trnH*, *ycf6-psbM*, *rpl32-trnL*, *trnL-F*, and *rbcL* regions, using the BI and ML methods. The tree topologies of the BI and ML trees were identical, and only the BI tree is shown (Fig. 6). A new haplotype (X), which occurred in all the *C. jeminaicum* individuals, was identified in the combined cpDNA dataset. The distribution of the *C. mongolicum* complex within the cpDNA tree could be separated into five to six regions that appear to reflect their geographical distribution. The first branch included sequences from six populations of the *C. mongolicum* complex (3, 4, 5, 9, 10, and 11) that were distributed in the west and northeastern regions of the Tengger Desert, where *C. arborescens* and *C. calliphysa*occurred sympatrically with these six populations. The second independent branch included sequences from four populations (14, 15, 16 and 17) from the Taklimakan Desert. The third independent branch included sequences from three populations (6, 7, and 8) from the Gurbantunggut Desert in the east of Xinjiang. Populations 12 and 13 comprised *C. mongolicum* complex samples from the Qaidam Desert that were distributed sympatrically with *C. ebinuricum*. Population 1 was the most phylogenetically distant from other populations, perhaps owing to its geographic isolation in the extreme north of Inner Mongolia. However, the new haplotype X of *C. jeminaicum* was separated from the above-mentioned branches of the *C. mongolicum* complex with strong support (Fig. 6, PP = 1, BS = 100%). Meanwhile, the other reference species of *Calligonum* (*C. ebinuricum*, *C. arborescens*, and *C. calliphysa*) did not form their own separate branches, but were interspersed within branches of the *C. mongolicum* complex (Fig. 6).

## Discussion

600 species names are known in *Calligonum*, but only 90 of these were recognized (Pavlov 1936; Baitenov and Pavlov 1960; Sergievskaya 1961; Drobov 1953; Kovalevskaya 1971; Liu and Yong 1985). Most of the new names occurring in *Calligonum* were subsequently ignored or merged into existing names (Pavlov 1936; Kovalevskaya 1971; Bao and Grabovskaya-Borodina 2003). Different taxonomists have controversial opinions on species delimitations in *Calligonum* (Soskov 2011; Zhang 2007; Sabirhazi et al. 2010; Abdurahman et al. 2012; Shi et al. 2016, 2017). For example, *C. rubescens* was treated as an independent species (Soskov 2011) by merging three species, *C. pumilum*, *C. alashanicum*, and *C. jeminaicum*. The taxonomical relationships of *C. pumilum*, *C. alashanicum*, and *C. mongolicum* have been clarified, with *C. pumilum* and *C. alashanicum* being merged into *C. mongolicum* (Shi et al. 2009). Additionally, *C. rubescens* was treated as a synonym of *C. mongolicum* (Shi et al. 2016). The relationship between *C. jeminaicum* and *C. mongolicum* was analyzed in the present study.

The morphological identification system, which has been used in the *C. mongolicum* complex (Shi et al. 2009), was employed here for phenotypic discrimination. Our results demonstrated that the fruit characters, which were confusing among members of the *C. mongolicum* complex, in addition to flower characteristics, can be used to distinguish *C. jeminaicum* from the *C. mongolicum* complex by statistical analysis. *C. jeminaicum* could be identified as a good species based on its morphology (Figs 2, 3).

DNA data are used as key evidence for taxonomical conclusions, and can also reveal the systematics among species or genera (Alvarez and Wendel 2003; Feliner and Rossello 2007). Molecular analyses of both nrDNA ITS and cpDNA sequence data (*trn*L-F, *mat*K, *atp*B-*rbc*L, *psb*A-*trn*H, *psb*K-*psb*L, and *rbc*L) fail to fully elucidate the taxonomical relationships within *Calligonum* (Tavakkoli et al. 2010; Sanchez et al. 2011; Sun and Zhang 2012; Li et al. 2014; Gouja et al. 2014), but some minor and reasonable taxonomical discrepancies among the controversial species group were resolved by combining the morphological and DNA data, for example, within the *C. mongolicum* complex (Shi et al. 2009, 2016, 2017) and between *C. trifarium* and *C. ebinuricum* (Abdurahman et al. 2012). The nrDNA tree, which combined nrITS and ETS data, suggested a lack of phylogenetic structure within the *C. mongolicum* complex, but it can be used to distinguish uncontested species in sect. Medusa, such as *C. arich*, *C. ebinuricum*, and *C. taklimakanense* (Shi et al. 2016). In the present study, *C. jeminaicum* formed a separate branch based on the nrITS and ETS data (Figs 4, 5), which is not consistent with the past or present occurrence of hybridization or interbreeding of *C. jeminaicum* with the *C. mongolicum*. Meanwhile, the cpDNA data were employed to confirm the taxonomic relationship of the *C. mongolicum* complex with *C. jeminaicum*. A new cpDNA haplotype (X) was identified in *C. jeminaicum*, and its separation from other haplotypes of the *C. mongolicum* complex and other species in sect. Medusa was well supported (Fig. 6). A high level of genetic diversity was also found in previous studies based on polymorphic cpDNA markers in the sect. Medusa(Wen et al. 2016b), especially in the *C. mongolicum* complex. The cpDNA information also revealed that the distributional ranges of some species in the *C. mongolicum* complex were geographically close or adjacent to each other (Figs 6). The distribution of genetic variation of the *C. mongolicum* complex in the Gurbantunggut Desert was consistent with its geographical signal, and the network analysis illustrated that genetic relationships in *Calligonum* formed a mesh pattern (Fig. 5). Compared to *C. mongolicum*, *C. jeminaicum* has a very narrow distribution with only one known population in the northwest of the Gurbantunggut Desert, which is also within the main distribution region of *C. mongolicum* (Mao and Pan 1986). It has been proposed that *C. jeminaicum* may contain only a small fraction of the total genetic variation present in its progenitor species in ancient Middle Asia (Sergievskaya 1961; Badria et al. 2007). This may have expanded the range of these xerophytes and allowed them to spread to other suitable habitats in the Jeminay area.

As an accepted name, *C. jeminaicum* has been confirmed as an endemic species which is found only within a relict area in the northwest of the Gurbantunggut Desert. *C. jeminaicum* has been on the brink of extinction over the past 40 years owing to the habitat of the only population being near the roads and the small number of individuals. Although the plants observed appeared to be healthy, the conservation of this plant species with an extremely small population (PSESP) (Wade et al. 2016) should receive appropriate attention in the future. As a result of a new policy framework, several national- and regional-level conservation strategies for China’s PSESPs are being implemented (Yang et al. 2015). For many of these species (Ren et al. 2012; Wang et al. 2017), the extinction of a population is irreversible; therefore, recognizing the immediate importance of these risk factors and understanding their interactions are crucial for developing future conservation plans (Volis 2016). The *in situ* conservation of the genetic diversity of *C. jeminaicum* for the long-term survival of this species requires a new management strategy that considers its reproductive biology and the future potential of hybridization/interbreeding. In the *ex situ* conservation of *C. jeminaicum*, special efforts are needed to ensure the isolation of genetic resources.

Since *Calligonum jeminaicum* is accepted as an independent species based on our new evidence; the threatened status of this species can be evaluated according to the International Union for Conservation of Nature (IUCN) Red List categories and criteria (IUCN 2012). This species was first collected by Zumei Mao together with Borong Pan from a single site near Jeminay, Xinjiang, China in the year 1979. It was described as a new species to science in 1984 (Mao 1984). Pan searched for this species in the original site and the surrounding area in 2008 but failed to find it. The first author (Wei Shi) searched for it again in 2013 in the Jeminay area and only a population with 8 mature (fruiting) individuals was found. No seeding or young individual was found in this population. No other collection or report of this species is available. Thus we evaluated *Calligonum jeminaicum* as Critically Endangered (CR) according to criteria D “Population size estimated to number fewer than 50 mature individuals” (IUCN 2012).
